# Impact of left ventricular ejection fraction and elevated filling pressures on mortality in mechanically ventilated patients in severe sepsis or septic shock

**DOI:** 10.1186/cc12162

**Published:** 2013-03-19

**Authors:** S Gillespie, J Pulido

**Affiliations:** 1 Mayo Clinic, Rochester, MN, USA

## Introduction

Myocardial dysfunction in septic shock is common and the presentation is broad. There are conflicting data regarding the prognostic implications of low left ventricular (LV) ejection fraction and elevated E/e' ratio on mortality in this patient population. We sought to assess the impact of LV dysfunction and elevated E/e' ratio on 30-day mortality in mechanically ventilated patients with severe sepsis or septic shock.

## Methods

Fifty-eight mechanically ventilated patients with severe sepsis or septic shock admitted from 1 August 2007 to 31 January 2009 were prospectively evaluated with transthoracic echocardiogram within 24 hours of admission. Left ventricular ejection fraction was assessed using the modified Simpson method as recommended by the American Society of Echocardiography. Normal LV function was defined as LVEF 40%. Mitral inflow pulsed wave Doppler of peak E waves and tissue Doppler imaging (TDI) of the septal mitral annulus peak velocities were measured, the E/e' ratio was obtained. Elevated LV filling pressures was defined as E/e' >15.

## Results

All cause 30-day mortality was 50% (*n *= 29). Forty-six patients (79%) had normal LV function. Forty-four (76%) patients had normal LV filling pressures. Patients who survived had lower E/e' ratio, (median E/e' 10.6 ± 4 vs. 13.6 ± 7, *P *= 0.1) but this was not statistically significant. However, when defined as E/e' >15, the mortality was 71%. On the contrary, patients with low LVEF had a mortality of 41% and LVEF was no different between survivors and nonsurvivors (55 ± 16 vs. 58 ± 14, *P *= 0.34).

## Conclusion

Myocardial dysfunction is a well-known entity in patients with septic shock. The clinical spectrum of this entity is broad, including LV, RV and diastolic dysfunction. Although the E/e' ratio has been a known prognostic indicator in other cardiac conditions, its role in these patients is less clear. This study demonstrated that when septal mitral annulus E/e' was >15, it was a better marker for mortality than LVEF in mechanically ventilated patients with severe sepsis or septic shock. Larger studies incorporating diastolic evaluation and TDI should be performed to further clarify this finding.
